# Clinical and Functional Results of Lateral Ulnar Collateral Ligament Repair for Posterolateral Rotatory Instability of the Elbow

**DOI:** 10.7759/cureus.53291

**Published:** 2024-01-31

**Authors:** Ahmad Almigdad, Sudhakar Challagundla, Tingshan Yan, Amar Malhas

**Affiliations:** 1 Department of Orthopedics, Royal Medical Services, Amman, JOR; 2 Department of Orthopaedics, Royal Cornwall Hospital, Truro, GBR; 3 Department of Orthopedics, Royal Berkshire NHS Foundation Trust, Reading, GBR; 4 Department of Orthopaedics, Royal Berkshire NHS Foundation Trust, Reading, GBR

**Keywords:** elbow dislocation, lateral ulnar collateral ligament., lateral collateral ligament, posterolateral rotary instability, elbow

## Abstract

Background

Posterolateral rotatory instability of the elbow arises from damage to the lateral ulnar collateral ligament (LUCL). While various methods exist for reconstructing or repairing the LUCL's attachment to the humerus, the most effective approach remains debatable. This study aims to assess the outcomes of directly repairing the LUCL when the injury occurs at the humeral attachment.

Methodology

This retrospective study, conducted at the Royal Berkshire Foundation Trust NHS hospital in Reading, UK, assessed outcomes through a review of 15 patients who underwent direct repair of the lateral ulnar collateral ligament between 2017 and 2022, evaluating a range of motion, the Mayo Elbow Performance Score, and the Nestor grading system.

Results

This study included nine males and six females, with an average age of 38.8 years. Most LUCL injuries arose from elbow dislocation (46.7%). The average follow-up period for patients was 26 months. At the final assessment, the mean Mayo Elbow Performance Score reached 99. According to the Nestor grading system, 12 patients achieved excellent results, and three had good outcomes. On average, there was an 11.3° loss of final extension and 5° of final flexion, yet achieving a comparable pronation-supination arch to the contralateral side.

Conclusion

Direct repair of the LUCL for elbow posterolateral rotary instability yielded excellent outcomes, obviating ligament reconstruction. Recognized as minimally invasive, it accelerates recovery, minimizes trauma, and offers a cost-effective procedure for managing instability.

## Introduction

Elbow stability is sustained through the combined influence of static and dynamic factors. The primary static constraints involve the osseous structure at the ulna-humeral-radius junction, the medial ulnar collateral ligament (MUCL), the lateral collateral ligament (LCL) complex, and the anterior capsule. Dynamic constraints encompass the neighboring muscular structures, and their associated fascial bands traverse the elbow joint, generating compressive forces to reinforce and safeguard the static stabilizers [1-3].

The elbow's lateral collateral ligament (LCL) complex comprises four primary components: the radial collateral ligament, the lateral ulnar collateral ligament (LUCL), the accessory LCL, and the annular ligament [4]. Elbow dislocations frequently disrupt the LCL complex, often concurrent with fractures, and often, the LUCL emerges from its humeral origin. This disruption can lead to chronic elbow instability, particularly identified as posterolateral rotatory instability, primarily due to damage in the LUCL segment of the LCL [5]. Surgical intervention commonly involves repairing the LCL, especially in cases where chronic instability symptoms persist or as part of the treatment for the elbow [6].

Posterolateral rotatory instability (PLRI) of the elbow, introduced by O’Driscoll et al. [7] in 1991, results from injury to the lateral ulnar collateral ligament (LUCL), leading to posterolateral subluxation or dislocation of the radius on the capitellum, yet not affecting the proximal radioulnar joint. It is commonly provoked by a traumatic event like elbow dislocation or significant valgus stress with axial loading [8]. PLRI can also emerge iatrogenically, like corticosteroid injections, lateral epicondylitis debridement, or due to ligament attenuation from chronic cubitus varus [9,10]. While O’Driscoll et al. initially highlighted LUCL injury as the primary cause, contemporary literature contests this, proposing that additional injury to other lateral elbow soft tissue structures is necessary for PLRI development [7,11]. Typically manifesting as pain and mechanical symptoms, managing PLRI usually involves surgical intervention for ligament repair or reconstruction [8].

Various techniques are described for repairing or reconstructing the humeral attachment of LUCL, although the best approach remains a matter of debate. In this study, we evaluate the surgical outcome of direct repair of LUCL where there has been an avulsion of the origin of the LUCL from the humerus.

## Materials and methods

This retrospective observational study was conducted at the Royal Berkshire Foundation Trust NHS hospital in Reading, United Kingdom. Electronic patient records were utilized to extract data on all patients who underwent direct repair of the Lateral Ulnar Collateral Ligament (LUCL) between 2017 and 2022. Patient information encompassed demographic characteristics, diagnoses, intraoperative findings, and recorded complications. The postoperative range of motion, Mayo Elbow Performance Score (MEPS), and Nestor grading system were utilized as the primary outcome measure.

Surgical technique

The surgery is performed under general anesthesia, with antibiotics administered during induction. The patient is positioned supine, with their arm resting on an arm table, and a high arm tourniquet is applied. A 5 cm longitudinal incision starts from the lateral epicondyle and extends distally over the radial head. The deep fascia is incised along this line, and the plane between the extensor carpi ulnaris and anconeus muscles is identified. The arm is kept in a pronated position to protect the posterior interosseous nerve. In case of trauma, an interval created by trauma can be used; otherwise, the radiocapitellar capsule is longitudinally incised, and the common extensor origin is left from the point of origin at the lateral epicondyle and lateral supracondylar ridge. The LUCL is identified, and the footprint is prepared.

**Figure 1:**
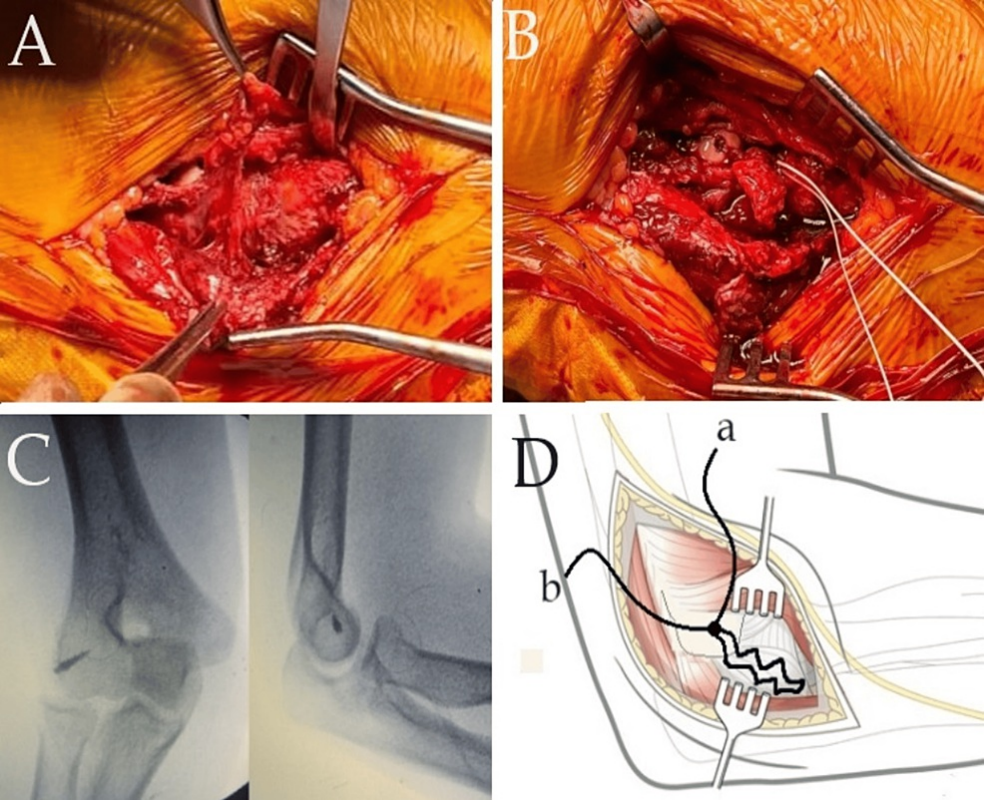
A) Intraoperative photo of humeral detachment of LUCL. B) Reattachment of LUCL to the humerus with a corkscrew anchor. ORIF of the radial head fracture is also seen in the image. C) Intraoperative fluoroscopic image showed the position of the corkscrew anchor with the anteroposterior and lateral views. D) A diagram demonstrating anchor positioning at the humeral insertion footprint of the LUCL. It exhibits two stitches passed through the ligament in an interdigitating manner while two additional limbs from the anchor (labeled as 'a' and 'b') are employed for repairing the common extensor origin and the anconeus tendon. LUCL: lateral ulnar collateral ligament; ORIF: open reduction and internal fixation

A 3.5 mm corkscrew is inserted at the LUCL's footprint on the lateral epicondyle. One suture is passed through the LUCL, starting from the tip toward the ulnar insertion and then passing back toward the tip in an interdigitating manner. The other suture is passed through the tip to prevent gapping. Subsequently, the knot is tightened with the elbow at a 45-degree angle of flexion. The suture is then used to repair the common extensor origin and repair the tendon of the anconeus with the aim of re-tensioning the anconeus (Figure 1). The distal and proximal intervals were then closed, utilizing a running or running-locking suture. The wound is closed, and a bulky dressing is applied, along with a sling for two weeks.

The patient is instructed on hand and wrist exercises and pronation and supination with the elbow at a 90° flexion starting the second day post-surgery. Between two to six weeks, gentle isometric and proprioception exercises for the elbow are initiated. From the sixth week onward, the patient begins a strengthening program throughout the range of movement, being cautious of varus strains on the elbow. The patient may return to light work that does not involve lifting after six weeks and heavy work after three months.

## Results

This study involved 15 patients who underwent direct repair for avulsion and/or attenuation of the LUCL over five years. All patients had an avulsion of the origin of the LUCL from the humerus, and the distal ligament was intact. Most patients were male (9 individuals), accounting for 60% of the cases. Among these cases, the right elbow was affected in 10 patients (66.7%). The mean age of patients at the time of injury was 38.8 years, ranging from 18 to 61 years (Table 1).

**Table 1:** Descriptive analysis of patients, N=15 RTA: road traffic accidents

	Total	%
Sex		
Male	9	60
Female	6	40
Dexterity		
Right	10	66.7
Left	5	33.3
Mechanism of injury		
RTA	2	13.3
Fall from a bike	2	13.3
Fall from a height	2	13.3
Fall from a standing height	6	40
No trauma	3	20
Associated injuries		
Elbow dislocation	7	46.7
Radial head fracture with instability	3	20
Chronic traumatic tear of the LUL	2	13.3
Chronic lateral epicondylitis	3	20

LUCL injury resulted from elbow dislocation in seven patients (46.7%) and was associated with radial head fracture in three patients (20%). Chronic traumatic tears of the LUL developed in two patients (13.3%) while in three patients (20%), the pathology was linked with chronic lateral epicondylitis.

Of the total, six patients received LUCL repair due to acute trauma, with an average time of 20 days between the injury and surgery. Another six patients underwent surgery due to elbow trauma sequelae, resulting in residual instability, with an average time to operation of 299 days. Additionally, three patients were operated on for chronic instability, with an average time of 715 days to the operation. The pathology in all patients was identified at the humeral attachment, and the ligament's quality was deemed suitable for repair.

The average follow-up period for patients was 26 months. At the final follow-up, the mean MEPS was 99. According to the Nestor grading system, 12 patients achieved excellent results while three had good outcomes. The mean loss of final extension was 11.3 ° ± 12 ° and final flexion was 5 ° ± 12.4 °, and patients achieved comparable pronation supination arch to the contralateral side. Furthermore, one patient underwent cubital tunnel decompression three months after the LUCL repair.

## Discussion

In 1991, O'Driscoll initially introduced the concept of posterolateral rotatory instability (PLRI) in the elbow, attributing the lateral ulnar collateral ligament (LUCL) as the primary cause of instability while emphasizing the intact nature of the annular ligament that sustains the stability of the proximal radioulnar joint [7]. However, recent literature has sparked debate regarding the LUCL's sole role in PLRI, with several anatomical studies suggesting the necessity of additional injuries to various lateral elbow structures, including the radial collateral ligament, parts of the annular ligament, and the common extensor mechanism. In some instances, the entirety of the lateral collateral ligament complex and the lateral joint capsule can detach from the humeral epicondyle as a unified tissue sheet. This displaced tissue, often positioned on the articular surface of the capitellum, obstructs the healing process of the ligamentous complex, ultimately leading to persistent posterolateral rotatory instability [12,13].

In PLRI, patients typically present with lateral elbow pain, notably during activities involving elbow extension and supination, such as rising from a chair by pushing up on armrests or performing a prone push-up motion. Mechanical symptoms like clicking, locking, or snapping are most pronounced at around 40° of flexion during arm extension [14].

Nonoperative methods often inadequately manage chronic posterolateral instability. Avoiding triggering positions and utilizing physical therapy or elbow bracing for mild symptoms can be challenging due to daily arm motion demands. Patients usually have a low tolerance for elbow braces. Specific conditions like open physes, significant elbow arthritis, or habitual dislocations may restrict surgery, benefiting from nonoperative measures. However, most chronic cases require surgical intervention [15]. Acute injuries with good ligament quality benefit from LUCL repair while chronic cases often need complex reconstructions. Correction of bone issues before surgery is crucial [16].

Ligamentous repair involves various approaches, primarily depending on the injury's nature. Acute injuries or those with adequate remaining ligamentous and capsular tissue may permit direct repair of the lateral ulnar collateral ligament through an open or arthroscopic technique. The injured ligament is reattached to the humeral attachment site via suture anchors or transosseous sutures, typically using a Bunnell suture method. Alternatively, suture anchors can be placed at the lateral epicondyle, securing the sutures in a horizontal mattress pattern [17,18]. However, chronic cases often necessitate ligamentous reconstruction due to poor tissue quality, requiring auto or allografts. Various autografts, such as palmaris longus, gracilis, and triceps fascia, have been discussed in the literature [19].

Several studies have contributed to understanding the clinical outcomes of operative treatments for PLRI of the elbow. O'Driscoll et al. published initial findings in 1991, where five patients underwent ligamentous repair or reconstruction, with favorable outcomes over a 15-30 month follow-up [7]. Nestor et al. reviewed 11 patients, noticing better outcomes with repair [20]. In comparison, Sanchez-Sotelo et al. expanded on this study with 44 PLRI patients, showing a higher incidence of residual instability in repair patients after a six-year follow-up [21].

Lee and Teo followed 10 PLRI patients, observing satisfactory results; however, their repairs were less successful than their reconstructions [22]. Olsen and Søjbjerg's study of 18 patients who underwent LUCL reconstruction highlighted success in most cases but noted decreased elbow extension strength after the procedure [23]. Savoie et al. analyzed 54 patients undergoing arthroscopic or open repair, finding comparable outcomes between the two approaches [24]. Jones et al.'s review of eight patients reported the resolution of instability post-reconstruction using a docking technique [25].

Anakwenze et al. conducted a systematic review, showcasing satisfactory outcomes for LUCL repair or reconstruction in a cohort of 130 patients from multiple studies. Most of the literature reflects favorable results with operative treatment for PLRI. Acute injuries of good tissue quality might benefit from direct repair while reconstruction using allograft is usually required for chronic PLRI to prevent recurrent instability. The choice between repair and reconstruction depends on the injury's chronicity and tissue quality, guiding the most suitable approach for patients' sustained stability [26].

In our study, encompassing 15 patients, all individuals underwent direct repair using an anchor to the lateral epicondyle. Remarkably, even in chronic cases, the tissues were reparable (as long as the avulsion is from the proximal origin), and no cases necessitated reconstruction. The average follow-up for patients was 26 months. Twelve patients demonstrated excellent outcomes while three showed good outcomes, as indicated by the Mayo Elbow Performance Score and Nestor grading system. All patients achieved functional movement, with a mean loss of 11.3° in final extension and 5° in final flexion, yet maintained a pronation-supination arch comparable to the contralateral side. Hence, our results align closely with those found in existing literature.

The study's limitations include its retrospective nature and a relatively small sample size of 15 patients, raising concerns about the generalizability of findings. The single-center design may limit diversity in cases and surgical techniques, impacting external validity. The 26-month follow-up, though valuable for short-term data, may not capture long-term complications. The absence of a comparative group hinders direct comparisons, making it challenging to draw definitive conclusions about the superiority of the direct repair approach. These limitations highlight the necessity for larger, multicenter prospective studies with extended follow-up to validate and generalize the study's findings.

## Conclusions

Managing acute and chronic elbow posterolateral rotary instability through direct repair of the LUCL, when feasible, yielded excellent to good clinical outcomes and eliminated the need for ligament reconstruction. Direct repair is considered a minimally invasive procedure compared to more extensive reconstruction, particularly when utilizing autograft. This approach expedites recovery and minimizes surgical trauma, underscoring a cost-effective and patient-centric approach to managing elbow instability.
